# A 3D finite element model to study the cavitation induced stresses on blood–vessel wall during the ultrasound-only phase of photo-mediated ultrasound therapy

**DOI:** 10.1063/5.0082429

**Published:** 2022-04-19

**Authors:** Rohit Singh, Xinmai Yang

**Affiliations:** Institute for Bioengineering Research and Department of Mechanical Engineering, University of Kansas, Lawrence, Kansas 66045, USA

## Abstract

Photo-mediated ultrasound therapy (PUT) is a novel technique utilizing synchronized ultrasound and laser to generate enhanced cavitation inside blood vessels. The enhanced cavitation inside blood vessels induces bio-effects, which can result in the removal of micro-vessels and the reduction in local blood perfusion. These bio-effects have the potential to treat neovascularization diseases in the eye, such as age-related macular degeneration and diabetic retinopathy. Currently, PUT is in the preclinical stage, and various PUT studies on *in vivo* rabbit eye models have shown successful removal of micro-vessels. PUT is completely non-invasive and particle-free as opposed to current clinical treatments such as anti-vascular endothelial growth factor therapy and photodynamic therapy, and it precisely removes micro-vessels without damaging the surrounding tissue, unlike laser photocoagulation therapy. The stresses produced by oscillating bubbles during PUT are responsible for the induced bio-effects in blood vessels. In our previous work, stresses induced during the first phase of PUT due to combined ultrasound and laser irradiation were studied using a 2D model. In this work, stresses induced during the third or last phase of PUT due to ultrasound alone were studied using a 3D finite element method-based numerical model. The results showed that the circumferential and shear stress increased as the bubble moves from the center of the vessel toward the vessel wall with more than a 16 times increase in shear stress from 1.848 to 31.060 kPa as compared to only a 4 times increase in circumferential stress from 211 to 906 kPa for a 2 *µ*m bubble placed inside a 10 *µ*m vessel on the application of 1 MHz ultrasound frequency and 130 kPa amplitude. In addition, the stresses decreased as the bubble was placed in smaller sized vessels with a larger decrease in circumferential stress. The changes in shear stress were found to be more dependent on the bubble–vessel wall distance, and the changes in circumferential stress were more dependent on the bubble oscillation amplitude. Moreover, the bubble shape changed to an ellipsoidal with a higher oscillation amplitude in the vessel’s axial direction as it was moved closer to the vessel wall, and the bubble oscillation amplitude decreased drastically as it was placed in vessels of a smaller size.

## I. INTRODUCTION

Therapeutic applications of biomedical ultrasound were first explored in the late 1920s.^1^ The focus of early studies was the thermal effect of high intensity ultrasound, which raises the temperature of a particular tissue above normothermic level.^2–4^ More recently, non-thermal effects of ultrasound such as radiation force, radiation torque, acoustic streaming, shock waves, and cavitation were explored.^5,6^ In the 1980s, shock wave lithotripsy (SWL) treatment, which uses the non-thermal effect of high intensity ultrasound to break kidney stones, was introduced in the United States.^7,8^ In the late 1980s, some studies found that the oscillation and collapse of vapor or air cavities played an important role in stone disintegration and could also induce vascular injuries during SWL treatment.^9–11^ Since then, many studies have been conducted to investigate the potential of ultrasound-induced cavitation for therapeutic applications, such as drug and gene delivery,^12–16^ blood–brain barrier opening,^17–20^ cell lysis,^21–23^ and thrombolysis.^24–26^

In the last 20 years, particularly, many studies have shown the potential of ultrasound-induced cavitation for the treatment of several medical conditions of blood vessels. Ultrasound-induced cavitation, including inertial and non-inertial cavitation inside blood vessels, produces higher than normal levels of shear and circumferential stresses, resulting in bio-effects on the blood vessel.^27–29^ Shear stress can produce a variety of bio-effects such as activation of ion channels,^30–32^ reversible perforation of the membrane,^33,34^ and cell detachment and lysis.^33,35^ Whereas the circumferential stress acts tangential to the vessel wall, it may produce an axial opening in the vessel wall, which causes vessel rupture and hemorrhage.^27,36,37^ Several studies have quantified cavitation induced circumferential stress with respect to vessel diameter, initial bubble size, ultrasound frequency, and amplitude.^20,38^

In order to better understand the cavitation effect in blood vessels under various ultrasound parameters, such as frequency, intensity, pressure, and material properties, numerical models have been developed to study the interaction between bubbles and the blood–vessel wall when ultrasound is applied. Several numerical models such as lumped parameter model, boundary element method, and finite element method (FEM) were used to study the resonance frequency of bubbles confined in a vessel. Qin and Ferrara^39^ used the lumped parameter model and Martynov *et al.*^40^ used the lumped parameter model coupled with Navier–Stokes equation to calculate the resonance frequency of a confined bubble. Qin and Ferrara^39^ found that for a microbubble confined inside a compliable vessel, the resonance frequency would decrease with increased vessel rigidity but increase with the decreased vessel size, while Martynov *et al.*^40^ showed that bubble confinement by an elastic vessel increases its natural frequency. Similarly, Wang *et al.*^41^ using the boundary element method coupled with FEM also found that the bubble resonance frequency increases and the oscillation amplitude decreases due to confinement by an elastic vessel. Martynov *et al.*^42^ developed an FEM based 2D model to study the bubble–vessel interaction and propagation of disturbances along the vessel wall for a bubble inside a narrow elastic tube. They concluded that the ratio of the excitation frequency to the resonance frequency was the most significant factor for propagating disturbances along the vessel wall. Hosseinkhah *et al.*^38,43^ using a 3D FEM model found that the resonance frequency of a contrast agent bubble would increase with increased vessel rigidity. Apart from the resonance frequency, numerical models were also used to calculate stresses induced on the vessel wall. Ye and Bull^44^ developed a 2D boundary element method to study the stresses induced on rigid and flexible tubes due to gas bubbles. They concluded that vessel wall stresses changed significantly with changes in vessel wall flexibility and initial bubble size to vessel size ratio. Miao *et al.*^45^ using the boundary element method coupled with FEM for the 2D model found that the decrease in vessel thickness, vessel radius, and acoustic frequency would increase circumferential stress. Gao *et al.*^46^ developed a 2D finite volume model to study the asymmetric bubble dynamics inside a deformable pseudoelastic vessel. They concluded that bubbles took an ellipsoidal shape due to vessel confinement and exerted higher stresses than a spherical shape. Recently, Hosseinkhah *et al.*^38,43^ developed a 3D FEM model coupled with the modified Rayleigh–Plesset equation to study contrast agent bubble dynamics inside a confined vessel. They calculated the circumferential and shear stress as functions of initial vessel size, initial bubble size, ultrasound frequency, and amplitude. All the above numerical studies found changes in the resonance frequency and a decrease in the oscillation amplitude due to vessel confinement as well as higher wall stresses due to bubble oscillation than normal physiological levels.

Recently, we have developed a novel technique to enhance ultrasound-induced cavitation inside blood vessels.^47–51^ In this technique, nanosecond laser pulses are synchronized with ultrasound bursts at the peak negative amplitude to induce enhanced cavitation. This novel technique utilizing concurrent laser and ultrasound is known as photo-mediated ultrasound therapy (PUT).^47,51,52^ Several PUT experimental studies have shown a reduction in local blood perfusion and removal of micro-vessels.^47,51^ These bio-effects have the potential to treat several medical conditions involving neovascularization such as cancer, age-related macular degeneration (AMD), and diabetic retinopathy (DR).^53–56^ Abnormal neovascularization in the choroid layer and retinal layer of the eye is often found in patients with AMD and DR, which are the leading causes of blindness in the U.S. Several treatment techniques, such as anti-VEGF (vascular endothelial growth factor) therapy, photodynamic therapy (PDT), and laser photocoagulation therapy (LPT), have been developed to remove the abnormal micro-blood vessels at the bottom of the eye to prevent blindness.^57–60^ Anti-VEGF is the most widely used technique in clinics. It prevents vision loss by reducing vascular permeability and neovascularization. However, anti-VEGF therapy is costly and inconvenient as it requires monthly hospital visits for injections. In addition, up to 50% of the patients will still become either completely or partially blind after 5 years of anti-VEGF therapy.^61^ In PDT, patients are required to avoid sun for several days after treatment due to the injection of photosensitizers during the treatment. Moreover, PDT may cause dye extravasation, photosensitivity reactions, transient visual disturbances, infusion-related back pain, and even choroidal infarction resulting in acute, severe vision loss.^62^ In LPT, the use of high laser energy can damage surrounding cells such as neurons, resulting in complications such as retinal atrophy, paracentral scotomas, sub-retinal neovascularization, and sub-retinal fibrosis.^63–65^ We have developed PUT to overcome the limitations of existing clinical techniques for the treatment of AMD and DR. PUT is completely non-invasive and particle-free as opposed to anti-VEGF therapy, which requires the injection of anti-VEGF agents, and PDT, which requires the injection of photosensitizers. In addition, PUT does not destroy the near-by tissues such as LPT as PUT uses low-fluence laser and low power ultrasound and only affects the region irradiated by both laser and ultrasound. Currently, PUT is in the preclinical stage, and several studies on *in vivo* rabbit eye models have shown that PUT can precisely remove micro-vessels in the eye.^51,66–68^

PUT does not require the use of ultrasound contrast agent bubbles; instead, the combined laser and ultrasound results in bubble nucleation by the photospallation effect.^69,70^ In photospallation, a strong thermal-elastic stress transient is produced in the region where light is absorbed.^69^ Moreover, for a cylindrical shaped absorber such as blood vessel, laser-induced thermal elastic pressure reflects at the vessel boundary and converges at the center region to produce significantly high rarefaction pressure.^71^ This laser-induced high rarefaction pressure when synchronized with ultrasound peak negative pressure results in cavitation.^49^ The induced cavitation is further enhanced by the subsequently applied ultrasound wave during PUT.

The cavitation process in PUT during an entire ultrasound burst has three major phases.^48^ During the first phase of PUT, laser induced photoacoustic waves synchronized with ultrasound peak negative pressure result in bubble nucleation. This nucleated bubble keeps on growing in size by the rectified diffusion process in the second phase of PUT. In the third and last phase of PUT, the bubble reaches a stable equilibrium radius and oscillates non-inertially around the equilibrium radius. In addition, the bubble slowly moves away from the center of the vessel toward the vessel wall in the last phase. During the entire ultrasound burst, the laser is irradiated only once on any of the first few ultrasound cycles at the peak negative amplitude to nucleate the bubble during the first phase. For the rest of the ultrasound burst, the formed bubbles are driven by ultrasound alone during the second and third phase of PUT.

In our previous work, we have studied cavitation inside the blood vessel during the first phase of PUT.^50^ We used a 2D axisymmetric FEM model with 50 and 100 nm bubbles in the center of 50–150 *µ*m vessels to simulate the interaction between blood vessels and oscillating bubbles during the first phase of PUT. The circumferential and shear stresses on the vessel wall induced by cavitation were calculated using the fluid-structure module in COMSOL Multiphysics 5.5 (Burlington, MA, USA). These stresses are thought to be responsible for cavitation induced bio-effects and vessel rupture. It was found that higher shear and circumferential stresses were induced on the vessel wall when the laser pulse was synchronized on the ultrasound burst, whereas no stresses were induced for the same ultrasound pulse alone.

In this work, our focus was to study the bubble dynamics and cavitation induced shear & circumferential stresses during the third or last phase of PUT. During ultrasound-induced bubble oscillation inside a blood vessel, beside radial oscillation, a bubble also translates toward the vessel wall. However, earlier numerical studies calculated bubble–vessel interaction by assuming that a bubble was present in the center of the vessel during the entire ultrasound pulse.^38–41,43–46^ These studies showed effects of changes in ultrasound parameters, bubble-–vessel sizes, and their mechanical properties on the resonance frequency, stresses induced on the vessel wall, the bubble oscillation amplitude, and the bubble shape. However, the effect of off-center bubbles on bubble–vessel interaction was only briefly considered by Hosseinkhah and Hynynen.^43^ The current study includes both on-center as well as off-center bubbles inside a blood vessel, and the confinement effect of the vessel on the bubble dynamics was considered.

In this study, we developed a 3D numerical model of an air bubble surrounded by blood and confined inside an elastic vessel to study the changes in the induced shear and circumferential stresses on the blood–vessel wall as a function of bubble movement toward the vessel wall. In addition, the effect of change in vessel radius on bubble dynamics of off-center bubbles and induced stresses on vessel wall were simulated. The model was solved by using the FEM in COMSOL Multiphysics 5.6 (Burlington, MA, USA). The model only used ultrasound waves for cavitation, and no laser effect was considered unlike our previous work^50^ as laser is not present in the last phase of PUT. The results of this study are not only limited to cavitation during the last phase of PUT but are applicable to any work involving ultrasonic induced cavitation inside the blood vessel.

## II. METHODS

**FIG. 1.**
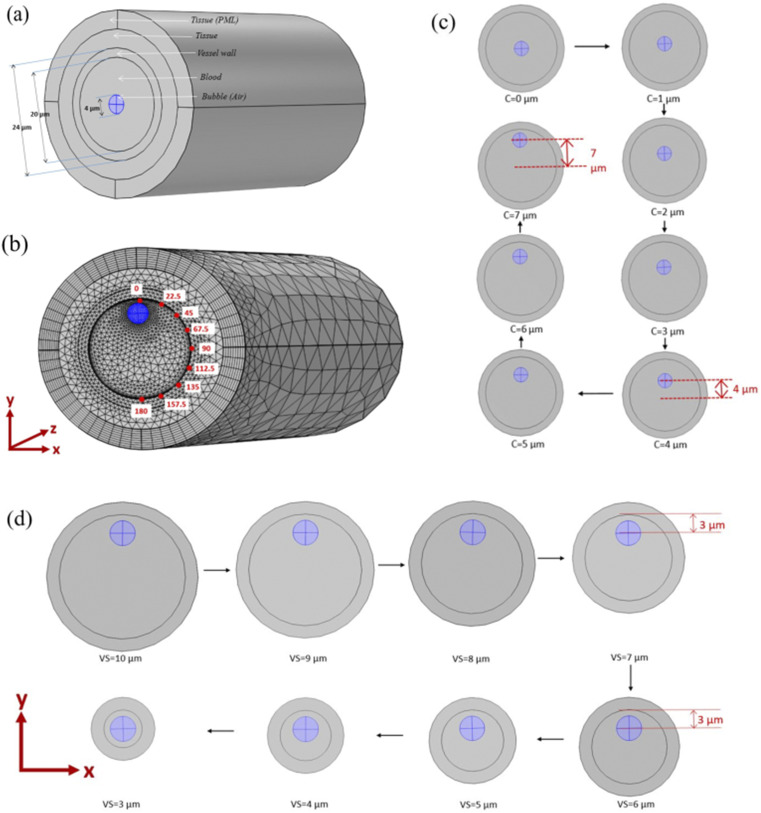
(a) A schematic of the bubble–blood–vessel–tissue 3D finite element model for a 2 *µ*m bubble in a 10 *µ*m vessel (PML: perfect matching layer). (b) Schematic of the meshed bubble–blood–vessel–tissue 3D finite element model of a 2 *µ*m bubble placed closed to the vessel wall in a 10 *µ*m vessel marked with directions on the vessel wall with respect to the vessel center. (c) A schematic of the bubble moving toward the vessel wall from the bubble center at 0 *µ*m distance from the vessel wall to 7 *µ*m distance in steps of 1 *µ*m in each step. (d) A schematic of a 2 *µ*m bubble placed in different size vessels with the minimum distance of 3 *µ*m between the vessel wall and bubble center. (VS: vessel size/bubble radius and C: bubble center distance from vessel center).

In our model, an air-filled microbubble surrounded by blood was placed inside a vessel; the vessel was further surrounded by a thick elastic tissue as shown in Fig. 1(a). Both air and blood were modeled as Newtonian fluids with air as compressible and blood as an incompressible fluid. The vessel and tissue were modeled as isotropic linear elastic solids. The study is divided into two major parts. In the first part, the changes in stresses induced on the vessel wall during movement of the bubble toward the vessel wall were simulated (presented in Sec. III C). In the second part, the effects of vessel radius change on induced stresses for off-center bubbles were simulated (presented in Sec. III D). Bubble radius of 2 *µ*m was used for all the simulations. The vessel radius of 10 *µ*m was used for the simulations in the first part and 3–10 *µ*m was used for the second part. The ultrasound wave with a frequency of 1 MHz and an amplitude of 130 kPa was used for all simulations. A smaller vessel radius of only 10 *µ*m was used to reduce the computational time of the model as the larger radius increases the computational time exponentially. A bubble size of 2 *µ*m, which is comparable to the vessel size of 10 *µ*m ,was selected for a larger interaction between the bubble and vessel wall. An ultrasound frequency of 1 MHz was used based on our previous numerical studies^48,50^ and experimental studies.^47,52^ Based on 1 MHz frequency and 2 *µ*m bubble radius, an ultrasound pressure amplitude of 130 kPa was selected as non-inertial cavitation with high oscillation amplitude take place at this pressure amplitude similar to the last/third phase of PUT. In addition, higher than a 130 kPa pressure amplitude results in inertial cavitation, which is not present in the third phase of PUT.

In the first part of the study, a total of eight different simulation cases were calculated with the bubble placed at different distances from the vessel center [Fig. 1(c)]. In the first simulation, the bubble was placed at the center of the blood vessel. In the second simulation case, the bubble was moved 1 *µ*m away from the vessel center, resulting in an off-center bubble. The bubble was moved 1 *µ*m away from the vessel center in consecutive simulation cases until its center reached a distance of 7 *µ*m from the vessel center in the eighth simulation case, as shown in Fig. 1(c). The first simulation case has an on-center bubble, while the rest seven simulation cases have an off-center bubble inside the blood vessel. Shear and circumferential stresses for each simulation case in different directions (0°, 22.5°, 45°, 67.5°, 90°, 112.5°, 135°, 157.5°, 180°) on vessel wall were calculated. In addition, centroid displacement of the bubble and its asymmetric ratio during oscillation were calculated for all cases.

In the second part of the study, a total of eight different simulation cases were calculated for different blood vessel sizes in each case. For every simulation case, the bubble was placed inside a vessel such that the minimum distance between the bubble center and the vessel wall was 3 *µ*m. In the first simulation, a vessel size of 10 *µ*m was considered with the bubble center at 7 *µ*m away from the vessel center. In the second simulation, the vessel radius size was reduced by 1 *µ*m resulting in a vessel of 9 *µ*m with an off-center bubble at a distance of 6 *µ*m away from the vessel center. The vessel radius was reduced by 1 *µ*m in each consecutive simulation until the bubble reached the center of the vessel in the eighth simulation. The eighth simulation has an on-center bubble, while the rest seven simulations have off-center bubbles inside a vessel. Shear and circumferential stresses for each simulation in different directions (0°, 22.5°, 45°, 67.5°, 90°, 112.5°, 135°, 157.5°, and 180°) on the vessel wall were calculated. In addition, centroid displacement of the bubble and its asymmetric ratio during oscillation were calculated for all simulation cases.

### A. Validation with the Keller–Miksis equation

As the first step, the Keller–Miksis equation was used to validate the model by comparing the bubble radius obtained from the Keller–Miksis equation and our 3D FEM model.^72^ The Keller–Miksis equation has the following form:

$$\begin{array}{ll} & \left(1-\frac{\dot{R}}{c}\right)R\ddot{R}+\frac{3}{2}\left(1-\frac{\dot{R}}{3c}\right){\dot{R}}^{2}\\ & \;=\frac{R}{\rho c}\frac{d}{dt}\left(P_{b}\right)+\frac{1}{\rho }\left(1+\frac{\dot{R}}{c}\right)\left(P_{b}-P_{\infty }-p\left(t+\frac{R}{c}\right)\right), \end{array}$$ (1)

$$P_{b}=\left(P+\frac{2\sigma }{R_{o}}\right){\left(\frac{R_{o}}{R}\right)}^{3k}-\left(\frac{2\sigma }{R}\right)-\left(\frac{4\mu \dot{R}}{R}\right),$$ (2)

where $R,\dot{R}$ and $\ddot{R}$ are the bubble radius, bubble velocity and bubble acceleration, respectively, c is the sound speed in blood, ρ is the blood density, *P*_*b*_ is the pressure in blood at the bubble–air interface, *P*_∞_ is the ambient blood pressure, μ is the blood viscosity, $p\left(t+\frac{R}{c}\right)$ is the ultrasound pressure, and *k* is the polytropic index of the process. The values of constants, ρ, *P*_∞_, *σ*, μ, and c for blood were assumed as 1055 kg/m^3^, 104.6 kPa, 0.072 N/m, 0.005 Pa s, and 1500 m/s, respectively, and the process was assumed isothermal (k = 1). The Keller–Miksis equation was derived from the Navier–Stokes equation and conservation of mass, the same equation COMSOL used to solve fluid dynamics.^72^ The Keller–Miksis equation does not include the confinement effect and assumes infinite blood around a spherically symmetric bubble, whereas a bubble confined inside a vessel has a smaller oscillation amplitude as compared to a free bubble and also changes its shape to ellipsoidal due to asymmetric oscillation. The 3D FEM model developed in this study includes the confinement effect due to the blood vessel. We found that the confinement effect is only prominent for a bubble with a radius more than 1/10th of the vessel radius. Therefore, validation with the Keller–Miksis equation was only performed for bubbles with radii no more than 1/10th of the vessel radius. In the current study, a bubble of 2 *µ*m in the center of a 20 *µ*m vessel resulting in a bubble to vessel radius of 1/10th was considered for the validation with the Keller–Miksis equation. In addition, the current 3D model was an extension of the 2D axisymmetric model used in our previous study.^50^ This 3D model has the same equations for the fluid domain and solid domain and same material properties and boundary conditions for the blood, vessel, and tissue domain as in our 2D axisymmetric model. Only the bubble domain fluid dynamics is different. In the 2D model, the bubble was assumed symmetric and its fluid dynamics was not solved using COMSOL, whereas in the 3D model, the bubble does not have symmetric constraints and its fluid dynamics was solved using COMSOL. Therefore, only the bubble displacement is validated for the 3D model by comparing it to the Keller–Miksis equation in this study, and the rest of the model was already validated in the 2D model.

### B. Fluid domain for blood during FEM analysis

Blood and air were modeled as a fluid domain and assumed as Newtonian. Blood was assumed as incompressible while air was assumed as compressible. The transient Navier–Stokes equation (3) and (5) and continuity equation (4) and (6) for an incompressible fluid (3) and (4) and compressible fluid (5) and (6) were used to solve the pressure and velocity fields in the blood domain and air domain,

$$\rho_{0}\frac{\partial \text{v}}{\partial t}+\rho_{0}\left(\text{v}\cdot \nabla \right)\text{v}=\nabla \cdot \left[-p\text{I}+\mu \left(\nabla \text{v}+{\left(\nabla \text{v}\right)}^{T}\right)\right]+\text{F},$$ (3)

$$\rho_{0}\nabla \cdot \left(\text{v}\right)=0,$$ (4)

$$\rho \frac{\partial \text{v}}{\partial t}+\rho \left(\text{v}\cdot \nabla \right)\text{v}=\nabla \cdot \left[-p\text{I}+\mu \left(\nabla \text{v}+{\left(\nabla \text{v}\right)}^{T}\right)\right]+\text{F},$$ (5)

$$\frac{\partial \rho }{\partial t}+\nabla \cdot \left(\rho \text{v}\right)=0,$$ (6)

where *ρ*_0_ and *ρ* are the density, *v* is the velocity vector, *p* is pressure, *μ* is blood viscosity, and *F* is the volume force vector. The blood viscosity was assumed to be 0.005 Pa s, and the blood density was constant and assumed to be 1055 kg/m^3^. The air viscosity was assumed to be 1.814 × 10^−5^ Pa s, and the blood density was calculated using Eq. (7),

$$\rho =\frac{P}{RT},$$ (7)

where *P* is the absolute pressure, R is the universal gas constant, and T is the absolute temperature. T was a constant and assumed to be 293 K, and R was 8.3145 J/(mol K).

### C. Solid domain for blood during FEM analysis

The vessel and tissue were modeled as solid domains and were assumed to be isotropic linear elastic solids. The deformation in vessel and tissue was given by Eq. (8),

$$\rho_{s}\frac{\partial^{2}\text{u}}{\partial t}=\nabla \cdot \sigma +{\text{F}}_{\text{v}},$$ (8)

where *ρ*_*s*_ is the solid density, *u* is the displacement vector, *σ* is the stress tensor, and F is the volume force vector. The vessel was given a Young’s modulus of 1.5 MPa and a density of 1070 kg/m^3^.^73^ The blood vessel was assumed to be surrounded by elastic muscle tissue with a Young’s modulus of 0.5 MPa and a density of 1055 kg/m^3^.^73^ Both solids were given Poisson’s ratio of 0.49.^73^

### D. Boundary conditions and FEM solution

The 3D FEM based numerical model was solved using the fluid-structure interaction module in COMSOL Multiphysics 5.6 (Burlington, MA, USA). An ambient pressure of 104.6 kPa was used, and an initial pressure of 72 kPa (2*σ*/*R*_*o*_) was given to the air domain to balance the surface tension of the bubble–blood interface. The perfectly matched layer (PML) was given to the exterior layer of tissue encompassing the whole model. The PML prevents reflection and absorbs all the waves by virtually stretching the tissue to make it as infinite tissue. The ends of the fluid and solid domains were given a symmetric boundary condition using Eq. (9) for the solid and Eqs. (10) and (11) for the fluid. Equation (9) sets the normal displacement to the solid boundary as zero. For the fluid, Eqs. (10) and (11) set the zero penetration of fluid and vanishing shear stresses. The symmetric boundary in the fluid and solid domains makes the model infinitely large in the horizontal direction,

$${\text{u}}_{\text{solid}}\cdot n=0,$$ (9)

$${\text{v}}_{\text{fluid}}\cdot n=0,$$ (10)

$$\mu \left(\nabla \text{v}+{\left(\nabla \text{v}\right)}^{T}\right)-\left(\mu \left(\nabla \text{v}+{\left(\nabla \text{v}\right)}^{T}\right)\cdot n\right)n=0.$$ (11)

At the blood and vessel interface, a fully coupled fluid–solid interaction boundary condition and no slip boundary condition were applied. The blood velocity (*v*_*fluid*_) and vessel displacement (*u*_*solid*_) at the blood and vessel interface are related by Eq. (12), and the relation between the stress tensor in the vessel (*σ*) and blood (Γ) is shown by Eq. (13). The blood velocity (*v*_*fluid*_) was equal to the wall velocity (*v*_*tr*_) at the blood–vessel interface due to the no-slip boundary condition as shown by Eq. (14). The pressure and viscous forces in blood as shown by Eq. (15) were applied as loads to the vessel using Eq. (13), which caused deformation of the vessel (*u*_*solid*_). In response, the deformation of the vessel caused changes in the blood domain using Eqs. (12) and (14),

$${\text{v}}_{\text{fluid}}=\frac{\partial u_{\text{solid}}}{\partial t},$$ (12)

σ⋅n=Γ⋅n, (13)

$${\text{v}}_{\text{fluid}}={\text{v}}_{tr},$$ (14)

$$\Gamma =\left[-p\text{I}+\mu \left(\nabla {\text{v}}_{\text{fluid}}+{\left(\nabla {\text{v}}_{\text{fluid}}\right)}^{T}\right)\right].$$ (15)

For the fluid domain (blood), a moving mesh based on the combination of Lagrangian formulation and Eulerian formulation, known as Arbitrary Lagrangian–Eulerian (ALE) mesh, was used.^74^ The Lagrangian formulation is based on the material coordinate system, and the Eulerian formulation is based on the spatial coordinate system. Generally, Lagrangian is used for simulating solids as it allows for moving boundaries, and Eulerian is used for simulating fluids as it does not allow moving boundaries. A combination of both, which allows for the movement of boundaries, was used to account for the movement of the vessel–blood interface and bubble–blood interface as a function of time. The Yeoh smoothing method, which is highly non-linear and uses minimum mesh deformation energy criteria, was used for ALE mesh deformation. It allows for larger displacement of boundaries and responds better to element distortion.

For meshing of the 3D model, tetrahedral elements were used. In the solid domain, quadratic Lagrange interpolation was used for the displacement field, and in the fluid domain, piecewise linear interpolation was used for velocity and pressure fields. The mesh was fine near the bubble–blood interface and vessel–blood interface and coarse away from the interface. The bubble–blood interface was first finely meshed. The blood, vessel, and tissue domains were meshed afterward in accordance with the mesh size at the bubble–blood interface. The bubble–blood interface was meshed with 622 elements as the meshed bubble volume (16.602 *µ*m^3^) was only 0.91% less than the actual bubble volume (16.755 *µ*m^3^), and the entire mesh had ∼60 000 elements. A boundary layer mesh was also applied in the blood domain at the blood–vessel interface. A time dependent PARDISO solver with a maximum time step of 3 ns was used for solving the model. The 3 ns maximum time step was selected based on the total computation time and accuracy of the model. A larger than 3 ns maximum time step will increase the error in bubble displacement significantly, while a maximum time step smaller than 3 ns will significantly increase the computational time but without significantly reducing the error in the bubble displacement.

After solving the 3D FEM-based model, the stresses induced on the vessel wall due to bubble oscillation were calculated using the following equations:

$$\sigma_{cr}=\frac{P_{i}{r_{i}}^{2}}{{r_{o}}^{2}-{r_{i}}^{2}}+\frac{{r_{i}}^{2}{r_{o}}^{2}}{r^{2}}\left(\frac{P_{i}}{{r_{o}}^{2}-{r_{i}}^{2}}\right),$$ (16)

$$\tau_{yz}=\mu \left(\frac{\partial v}{\partial z}+\frac{\partial w}{\partial y}\right),$$ (17)

where *σ*_*cr*_ is the circumferential stress and *τ*_*yz*_ is the shear stress. For the circumferential stress, the vessel was assumed as a thick cylinder such that *r*_*i*_ and *r*_*o*_ are the inner and outer radius of the vessel, respectively, and *P*_*i*_ is the pressure on the inner radius of the vessel. The shear stress is proportional to the blood velocity gradient ($\frac{\partial v}{\partial z},\frac{\partial w}{\partial y}$) on the vessel wall, where v is the fluid velocity in y direction and w is fluid velocity in z direction.

## III. RESULTS

### A. Validation of the model

**FIG. 2.**
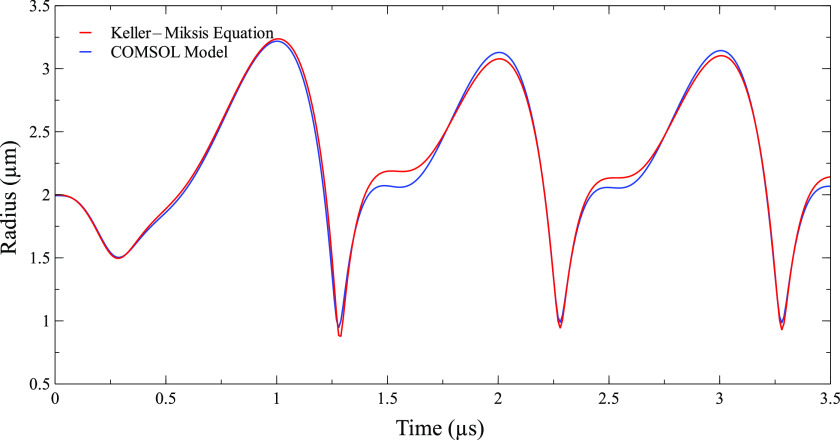
Radius of a 2 *µ*m bubble calculated from the Keller–Miksis equation and our 3D FEM model for a peak ultrasound pressure of 130 kPa and an ultrasound frequency of 1 MHz. A vessel radius of 20 *µ*m was used in the COMSOL model to reduce the confinement effect.

First, the model was validated by comparing it with the Keller–Miksis equation. To compare our model with the Keller Miksis equation, we placed a 2 *µ*m bubble inside a 20 *µ*m vessel such that the initial bubble size was 1/10th of the vessel size to keep the confinement effect minimum. The bubble radius from the Keller–Miksis equation and our COMSOL model are compared in Fig. 2. The bubble radius from our model is very close to the bubble radius from the Keller–Miksis equation with a root mean square deviation of 4.88%.

### B. Circumferential and shear stress

**FIG. 3.**
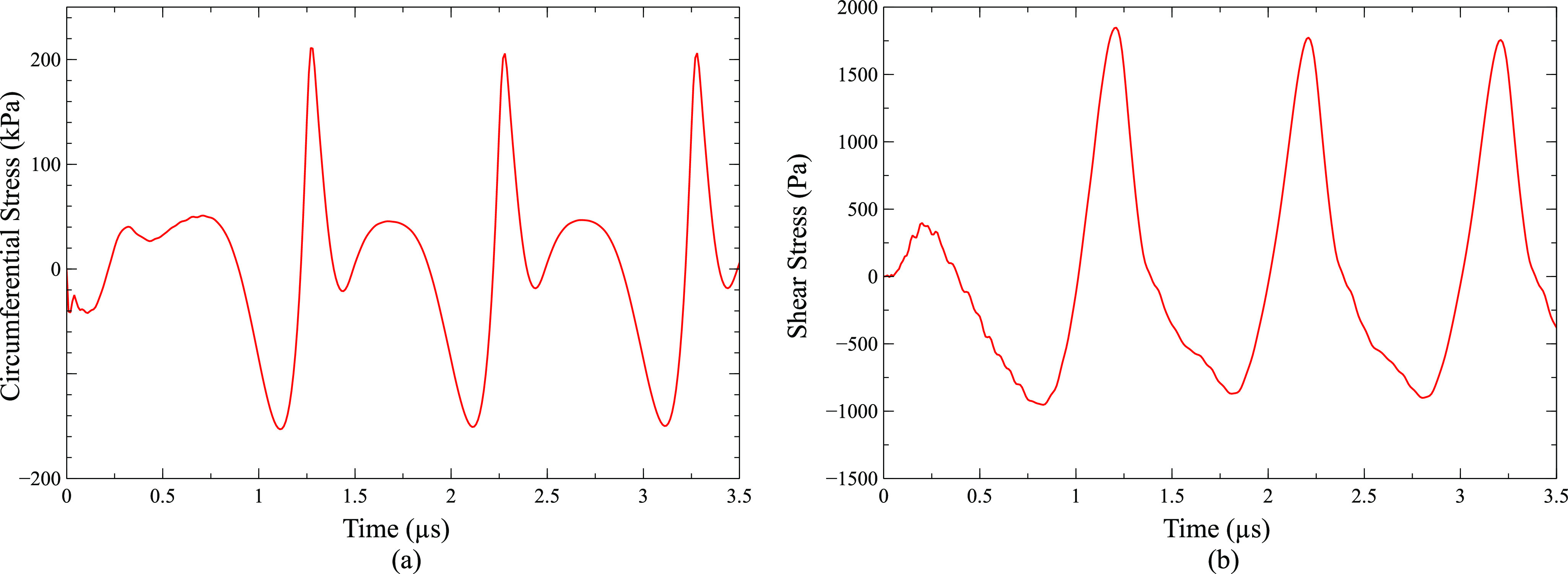
(a) Circumferential stress and (b) shear stress due to the oscillation of a 2 *µ*m bubble in the center of a 10 *µ*m vessel. (Peak ultrasound pressure = 130 kPa and ultrasound frequency = 1 MHz).

Figure 3 shows the circumferential and shear stress on the vessel wall due to oscillation of a 2 *µ*m bubble in the center of a 10 *µ*m vessel when an ultrasound wave of 130 kPa pressure was applied. The maximum circumferential stress of 211 kPa at 1.27 *µ*s and the minimum circumferential stress of −153 kPa at 1.13 *µ*s were observed. The maximum shear stress of 1848 Pa at 1.21 *µ*s and the minimum shear stress of −953 Pa at 0.83 *µ*s were obtained. The maximum circumferential stress on the vessel wall was obtained at a point directly above the bubble center similar to our previous study^50^ and other studies,^38,43,46^ while the maximum shear stress on the vessel wall was found at a point 4 *µ*m away from the point directly above the bubble center similar to our previous study^50^ and other studies.^38,43^ The stress oscillation repeated its pattern with the bubble oscillation. The maximum circumferential stress and maximum shear stress during the entire cycle will be used for comparison in further results.

### C. Bubble movement toward vessel wall

#### 1. Maximum circumferential and shear stress

**FIG. 4.**
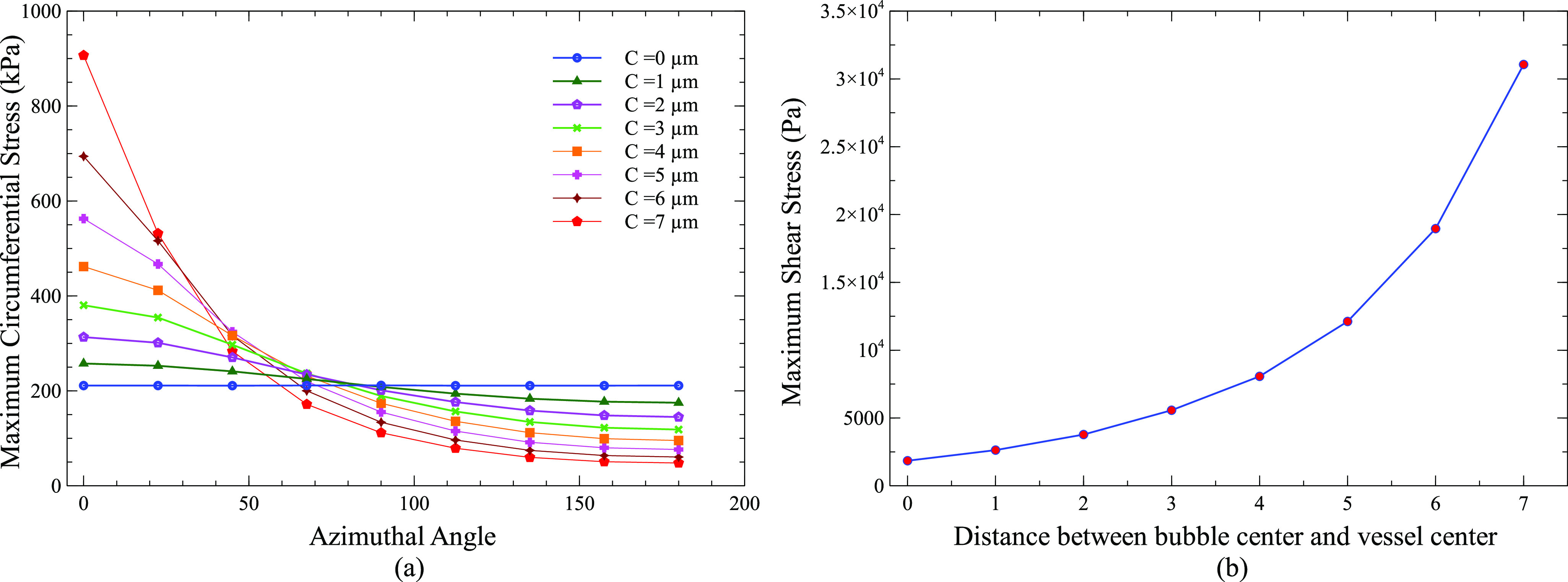
(a) Maximum circumferential stress as a function of the azimuthal angle for a 2 *µ*m bubble placed at 0, 1, 2, 3, 4, 5, 6, and 7 *µ*m away from the vessel center. (b) Maximum shear stress as a function of the distance between the bubble center and vessel center. (Peak ultrasound pressure = 130 kPa, ultrasound frequency = 1 MHz, vessel radius = 10 *µ*m, and C = distance between the bubble center and vessel center).

Figure 4(a) shows the maximum circumferential stress on the vessel wall as a function of the azimuthal angle for a bubble placed at 0–7 *µ*m away from the center of the vessel. The azimuthal angle here was defined with respect to the vessel center instead of the bubble center. The azimuthal angles represent different directions on the vessel wall as shown in Fig. 1(b). When the bubble was placed in the center of the vessel (C = 0 *µ*m), a uniform maximum circumferential stress of 211 kPa was obtained in all directions (0°–180°) on the vessel wall due to the constant distance between the bubble surface and the vessel wall in all directions. However, as the bubble was moved away from the vessel center, the maximum circumferential stress increased on the vessel wall between 0° and 50° with a maximum increase at 0° and decreased between 90° and 180° azimuthal angle with the least value at 180°. For a bubble at 7 *µ*m away from the vessel center, a maximum circumferential stress of 906 kPa was induced at 0° and 48 kPa at 180°. Similarly, the maximum increase in shear stress was at 0°, and the maximum decrease was at 180° as the bubble was moved toward the vessel wall. The maximum shear stress at 0° was 1.848 kPa for a bubble in the center of vessel and 31.060 kPa for a bubble 7 *µ*m away from the vessel center.

#### 2. Microstreaming

**FIG. 5.**
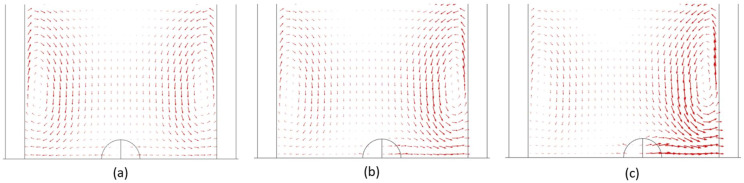
Blood velocity vector representing microstreaming at 2.5 *µ*s for a bubble placed (a) 0 *µ*m away from the vessel center, (b) 1 *µ*m away from the vessel center, and (c) 2 *µ*m away from the vessel center. (Peak ultrasound pressure = 130 kPa, ultrasound frequency = 1 MHz, and vessel radius = 10 *µ*m).

Figure 5 shows the velocity vectors at 2.5 *µ*s for bubbles at distances of 0–2 *µ*m away from the vessel center. The microstreaming due to bubble oscillation was clearly visible on both sides of the bubble (0° and 180°). In Fig. 5(a), the velocity vector and microstreaming magnitude were the same on 0° and 180°. However, in Fig. 5(b), for a bubble center 1 *µ*m away from a vessel center, the microstreaming magnitude was greater at 0° and smaller at 180°. Similarly, in Fig. 5(c), the microstreaming magnitude at 0° was much greater than that at 180°. This increase in the microstreaming magnitude at 0° and the decrease at 180° with bubble movement toward the vessel wall were the reasons for the increase in max shear stress at 0° as shown in Fig. 4(b).

#### 3. Centroid displacement and asymmetry ratio

**FIG. 6.**
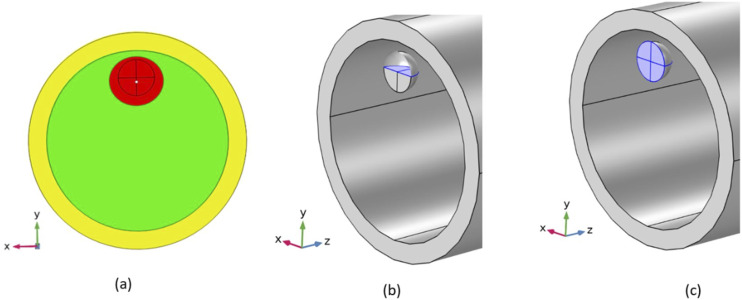
(a) A schematic illustrating the bubble centroid displacement away from the vessel wall as a bubble is expanding more away from the vessel wall. (b) Bubble cross section area parallel to the vessel axis (z) in blue color. (c) Bubble cross section area perpendicular to the vessel axis (z) in blue color.

**FIG. 7.**
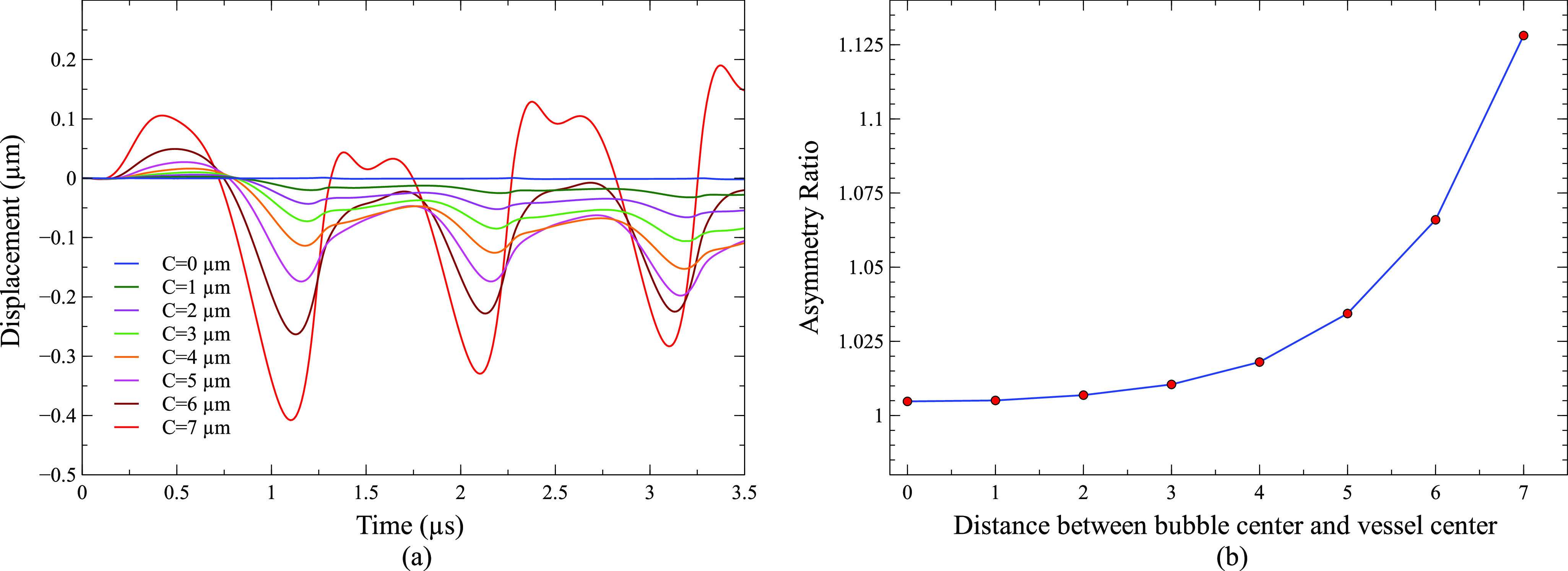
(a) Centroid displacement of a 2 *µ*m bubble placed at 0–7 *µ*m away from the vessel center. (b) Asymmetric ratio as a function of the distance between the bubble center and vessel center (peak ultrasound pressure = 130 kPa, ultrasound frequency = 1 MHz, vessel radius = 10 *µ*m, and C = distance between the bubble center and vessel center).

Figure 7 shows the asymmetricity of a bubble during oscillation using centroid displacement [Fig. 7(a)] and asymmetric ratio [Fig. 7(b)]. The bubble centroid displacement in Fig. 7(a) represents the displacement away from the vessel wall along the direction of bubble movement [y direction in Fig. 6(a)]. The centroid displacement signifies the bubble asymmetricity and overall displacement of a bubble toward and away from the vessel wall. Positive displacement signifies the centroid displacement toward the vessel wall, and negative displacement signifies the centroid displacement away from the vessel wall. Figure 6(a) shows the centroid displaced by 0.38 *µ*m away from the vessel wall at 1.05 *µ*s when a bubble was placed at a distance of 7 *µ*m away from the vessel center (C = 7 *µ*m). Figure 7(a) shows the centroid displacement of a 2 *µ*m bubble placed at 0–7 *µ*m away from the vessel center. The centroid displacement is 0 *µ*m for a bubble placed in the center of the vessel. However, centroid displacement increased when a bubble moved toward the vessel wall. In Fig. 7(a), after reaching the maximum compression point at 0.28 *µ*s, the bubble starts to expand more toward the vessel wall (toward 0°) initially causing the centroid displacement toward the vessel wall. In the later expansion phase, the bubble expands more toward 180°, resulting in centroid displacement away from the vessel wall. The same pattern of centroid displacement was obtained for all the off-center bubbles.

The asymmetric ratio is the ratio of the maximum area of the bubble cross section parallel to the vessel axis as shown in Fig. 6(b) to the maximum area of the bubble cross section perpendicular to the vessel axis as shown in Fig. 6(c). The asymmetric ratio signifies the asymmetry in bubble oscillation along the vessel axis (z-direction) and perpendicular to the vessel axis (x-y plane). As the bubble moved toward the vessel wall, the asymmetric ratio increased, which meant the bubble oscillation amplitude was more in the vessel axial direction as compared to the vessel transverse direction. The increase in the asymmetric ratio was due to increased confinement as the bubble was moved closer to the vessel wall. The asymmetric ratio was 1.005 even when it was placed in the center of the vessel due to vessel confinement.

### D. Bubble inside different vessel sizes

#### 1. Bubble volume

**FIG. 8.**
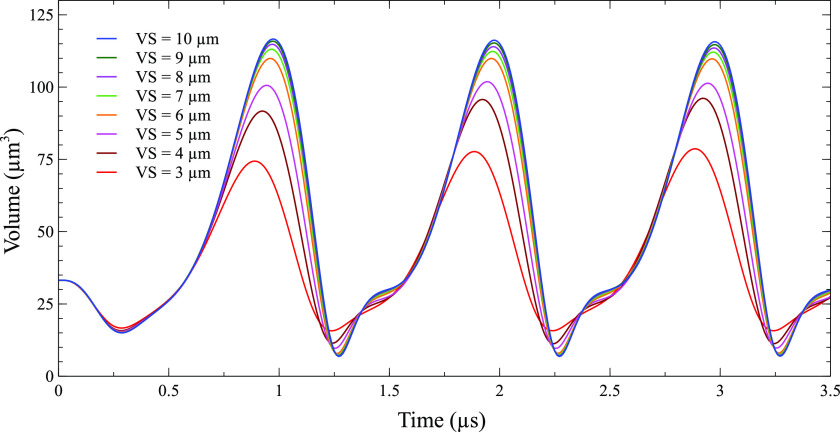
Volume of a 2 *µ*m bubble inside different size vessels. The minimum distance of 1 *µ*m between the bubble surface and vessel wall is maintained in all cases. (Peak ultrasound pressure = 130 kPa, ultrasound frequency = 1 MHz, and VS: vessel size/vessel radius).

Figure 8 shows the bubble volume of a 2 *µ*m bubble placed inside a vessel of size 10, 9, 8, 7, 6, 5, 4, and 3 *µ*m such that the minimum distance between the bubble center and vessel wall is 3 *µ*m in each case [Fig. 1(d)]. The bubble volume oscillated between the maximum of 116.63 *µ*m^3^ and the minimum of 6.82 *µ*m^3^ with an equilibrium volume of 33.51 *µ*m^3^ for the 2 *µ*m bubble inside a 10 *µ*m vessel when an ultrasound pressure of 130 kPa was applied. When the same bubble was placed in a 9 *µ*m vessel, its volume oscillated between the maximum of 115.9 *µ*m^3^ and the minimum of 6.89 *µ*m^3^ with the same ultrasound parameters. The amplitude of bubble oscillation decreased as the vessel size was reduced. For a bubble inside a 3 *µ*m vessel in the last case, the bubble reached a maximum volume of 78.65 *µ*m^3^ and a minimum volume of 15.71 *µ*m^3^ only. The bubble oscillation amplitude decreased due to the increase in the confinement effect of the vessel when the vessel size decreased. In case of the 10 *µ*m vessel, the bubble center was 3 *µ*m away from the vessel wall at a 0° azimuthal angle, whereas for the bubble inside a 3 *µ*m vessel, its center was 3 *µ*m away from the vessel wall in all directions (0° −180°).

#### 2. Maximum shear and circumferential stress

**FIG. 9.**
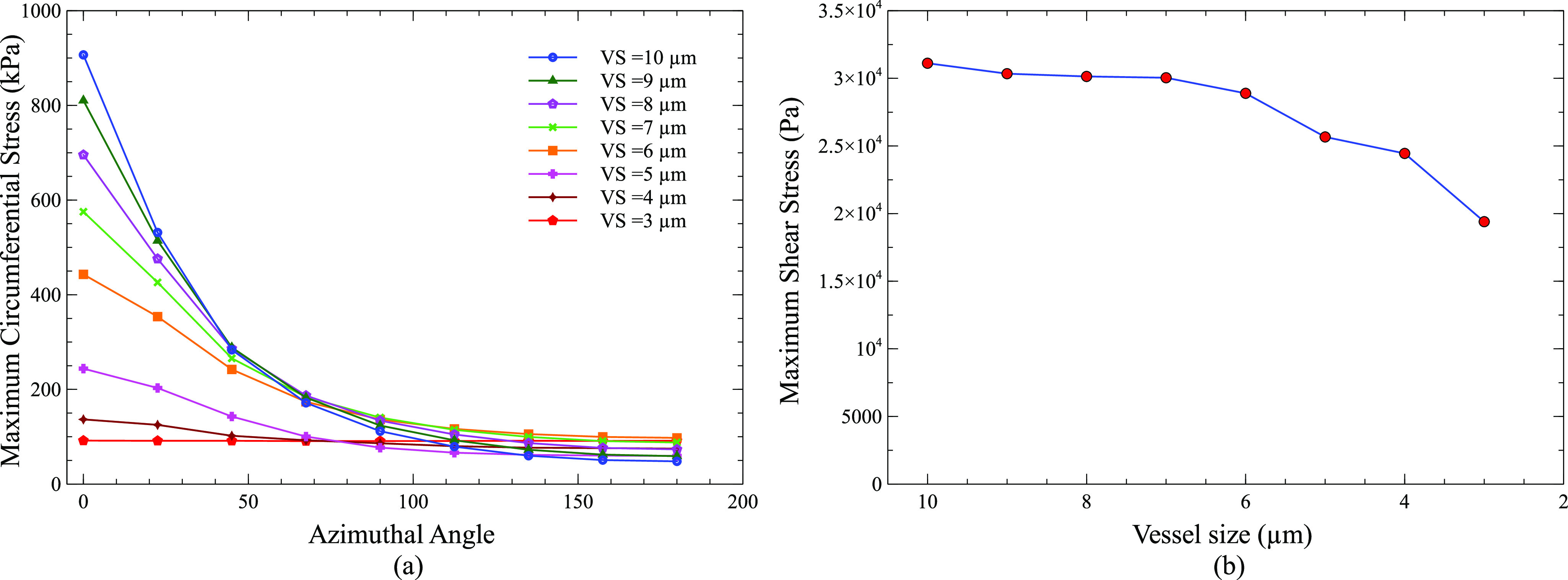
(a) Maximum circumferential stress as a function of the azimuthal angle for a 2 *µ*m bubble inside different size vessels. The minimum distance of 1 *µ*m between the bubble surface and vessel wall is maintained in all cases. (b) Maximum shear stress as a function of the vessel size. (Peak ultrasound pressure = 130 kPa, ultrasound frequency = 1 MHz, and VS: vessel size/vessel radius).

Figure 9(a) shows the maximum circumferential stress on the vessel wall as a function of the azimuthal angle for a 2 *µ*m bubble placed inside vessels of size 10^−3^ *µ*m. When a bubble was placed inside a 10 *µ*m vessel, the maximum circumferential stress at 0° was 906.67 kPa and 48 kPa at 180°. The maximum circumferential stress at 0° decreased as the vessel size decreased despite the same distance between the bubble and vessel wall in 0° direction. It decreased from 906.67 kPa for a 10 *µ*m vessel to 91.85 kPa for a 3 *µ*m vessel (90% decrease). This decrease was mainly due to the decrease in bubble amplitude oscillation. The maximum circumferential stress at 180° did not change much with changes in the vessel size. In addition, the maximum circumferential stress became more uniform along different directions between 0° and 180° as the vessel size was reduced or a bubble moved toward the vessel center.

Similarly, the maximum shear stress at 0° also decreased as the vessel size was reduced. However, the maximum shear stress only decreased from 31.116 to 19.4 kPa (38% decrease). The relatively small decrease in shear stress as compared to circumferential stress might be because the distance between the vessel wall and bubble surface was set as a constant in all the cases. Earlier study also pointed out that shear stress was more dependent on the distance between the vessel wall and bubble surface, while circumferential stress was more dependent on the bubble amplitude.^43^

#### 3. Centroid displacement and asymmetry ratio

**FIG. 10.**
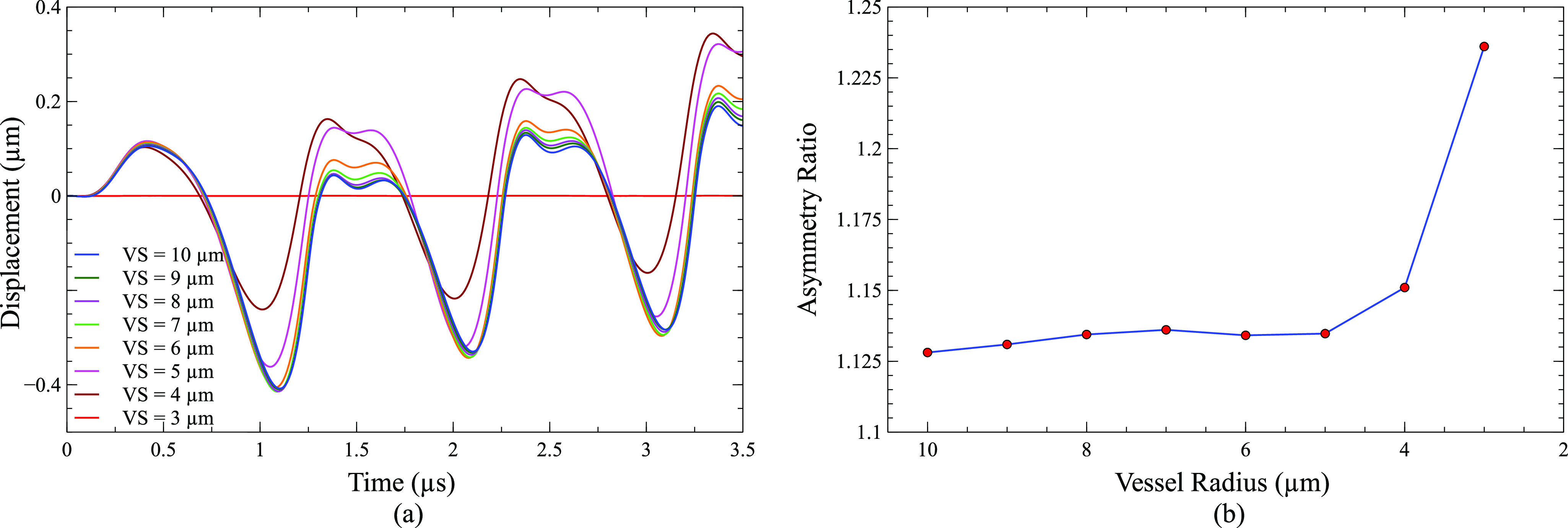
(a) Bubble centroid displacement of a 2 *µ*m bubble inside different size vessels. The minimum distance of 1 *µ*m between the bubble surface and vessel wall is maintained in all cases. (b) Asymmetric ratio as a function of vessel radius. (peak ultrasound pressure = 130 kPa, ultrasound frequency = 1 MHz, vessel radius = 10 *µ*m, and VS: vessel size/vessel radius).

Figure 10 shows the asymmetricity of the bubble during oscillation when it was placed in vessels of different sizes. The centroid displacement pattern was the same in each case as the minimum distance between the bubble and vessel wall was the same. However, the overall shift in the bubble centroid toward the vessel wall increased as the vessel size decreased. This was because when the bubble surface was getting closer to the vessel wall in the 180° direction, there was less space for a bubble to move in that direction. The asymmetric ratio depends upon the combination of the bubble amplitude and vessel confinement. Overall, the asymmetric ratio in bubble oscillation increased due to the increase in confinement. However, the asymmetric ratio decreased when the vessel radius was 6 *µ*m. This could possibly be due to a greater decrease in the bubble oscillation amplitude.

## IV. DISCUSSION

The normal shear stress inside the blood vessel has been determined using various theoretical and experimental studies. One study using large arteries with the diameter in range from 2.7 to 8.5 mm found peak and mean shear stress to be less than 5.5 and 1.6 Pa.^75^ Another study measured mean shear stress to be less than 5 Pa in arterioles larger than 15 *µ*m diameter of rabbits, cats, and rats.^76^ The theoretical calculation assuming constant shear stress along the arterial tree yielded a result of 1.5% ± 50% Pa.^75,76^ A study by combining theoretical simulation and experimental measurement estimated shear stress to be more than 10 Pa in capillaries smaller than 10 *µ*m in diameter.^77^ Based on these studies, the normal shear stress inside a blood vessel is less than 10 Pa in large vessels and may slightly exceed 10 Pa in small capillaries. In this study, the calculated shear stress induced by cavitation was in the kilo-pascal range with the lowest value of 1.848 kPa to highest value of 31 kPa. This magnitude of shear stress is significantly higher than the physiological level of shear stress; such high shear stress might be responsible for enhanced bio-effects during PUT.

The circumferential stress at which the blood vessel ruptures has also been measured in various studies. For the measurement of circumferential stress at vessel rupture, the fluid pressure inside the blood or water-filled vessel was slowly increased until rupture. The frog mesenteries were found to rupture at circumferential stress of 800 kPa^37^ while the rabbit capillary ruptured at 80 kPa.^78^ The abdominal aortic aneurysm ruptured at circumferential stress ranging from 200 to 2000 kPa depending upon the vessel diameter, thickness, and other properties.^79^ In this study, the highest value of calculated circumferential stress was 906 kPa when a bubble was very close to the vessel wall, whereas the lowest calculated value was 48 kPa for a bubble present in the center of a 3 *µ*m vessel. Based on the calculated values, circumferential stress might cause rupture only when a bubble is very close to the vessel wall.

The calculated values of shear stress and circumferential stress in this study depended mainly on two factors, the amplitude of bubble oscillation and the distance between a bubble and the vessel wall. As the bubble was moved toward the vessel wall, circumferential stress and shear stress increased dramatically (Fig. 4). When a bubble in the center of a vessel was moved toward the vessel wall by 1 *µ*m in a consecutive simulation case until it reached 7 *µ*m from the vessel center [Fig. 1(c)], the distance between the bubble and the vessel wall decreased drastically in the 0° direction, but the bubble oscillation amplitude only decreased slightly. The bubble placed in the center of a vessel oscillated between a maximum volume of 131.96 *µ*m^3^ and a minimum volume of 4.5385 *µ*m^3^, while the bubble placed at a distance of 7 *µ*m from the vessel center oscillated between a volume of 116.63 and 6.8172 *µ*m^3^ (Fig. S1). Therefore, the main factor for the increase in circumferential and shear stress in this case was the decreased distance between the vessel wall and the bubble. The increase in shear stress was more than 16 times, while that of circumferential stress was only 4 times of its initial value at the center of the vessel (Fig. 4). The shear stress increased due to increased microstreaming in the 0° direction (Fig. 5). A greater increase in shear stress is beneficial as it produces more bio-effects on the vessel wall during PUT, while a smaller increase in circumferential stress is useful as it reduces the chance of vessel rupture during treatment. When the vessel radius was decreased but the distance between the bubble and vessel wall remained the same in 0° direction [Fig. 1(d)], the shear stress and circumferential stresses decreased significantly (Fig. 9). The shear stress decreased to ½ times of its initial value, and the circumferential stress decreased to 1/15 times. The decrease in stresses in this case was mainly due to the reduced bubble oscillation amplitude because the oscillation amplitude decreased significantly with the decreased vessel size (Fig. 8), while the distance between the vessel wall and bubble remained the same in 0° direction. The increase in shear stress was greater than circumferential stress when the distance between the bubble and vessel wall was the main factor for changes in stress [Figs. 4 and 1(c)]. On the other hand, the decrease in circumferential stress was greater than the decrease in shear stress when bubble oscillation amplitude was the main factor for changes in stress [Figs. 1(d), 8, and 9]. This led us to the conclusion that shear stress is more dependent upon the distance between a bubble and vessel, while a change in bubble oscillation amplitude has a greater effect on circumferential stress. These findings are similar to an early study where circumferential and shear stress were calculated for bubbles placed inside the center of different size vessels.^43^

Apart from the changes in induced stresses on the vessel wall, the bubble shape, which was initially assumed to be spherical, also changed when a bubble was moved closer to the vessel wall and when it was placed inside vessels of different sizes. When a bubble was moved closer to the vessel wall, the bubble oscillates more in the vessel’s axial direction as compared to the lateral direction [Fig. 7(b)], resulting in an ellipsoidal shape. In the lateral direction, the oscillation amplitude was less as it was limited by vessel confinement while the axial direction was free of confinement. The bubble oscillation amplitude was higher by 0.5% in the axial direction than lateral direction when it was placed in the center of vessel, whereas it was 12.5% when it was placed 7 *µ*m away from vessel center [Fig. 7(b)]. The difference between the ellipsoid major (axial direction) and minor axis (lateral direction) increased as a bubble was moved toward the vessel wall. The bubble oscillation in the lateral direction also became asymmetric as it was moved away from the vessel center [Fig. 7(a)]. It expanded more in the 0° direction in the initial expansion phase and in the 180° direction in the lateral expansion phase, resulting in a non-uniform ellipsoid cross section area [Fig. 6(a)]. The reason for this lateral asymmetricity was non-uniform vessel confinement in the 0°–180° direction. The vessel confinement effect increased in the 0° direction and decreased in the 180° direction while a bubble was moved closer to the vessel wall, which resulted in an increase in lateral asymmetricity. In addition, a bubble overall displaces toward the vessel wall with each ultrasound cycle, and the overall displacement increased as the bubble was moved closer to the vessel wall.

In case where a bubble was placed inside vessels of different sizes while maintaining a fixed distance from the vessel wall in the 0° direction [Fig. 1(d)], almost similar results were obtained for all the cases except when the bubble was in the center of a 3 *µ*m vessel (Fig. 10). There was a slight change in overall bubble displacement toward the vessel wall as the vessel size was reduced to 5 *µ*m. This change was due to a significant decrease in the bubble oscillation amplitude (Fig. 8). When the vessel size was reduced to 3 *µ*m while maintaining the same distance in the 0° direction, the bubble reached the center of the vessel. For a bubble in the center of a vessel, the confinement is same in all the directions, which resulted in no lateral asymmetricity and no bubble displacement in lateral direction.

The bubble shape will change remarkably without any significant change in its oscillation amplitude as it moves closer to the vessel wall. Apart from the increased oscillation amplitude in the axial direction, the bubble will be asymmetric in the lateral direction. The simulation also shows that for an off-center bubble, the bubble–vessel dynamics force the bubble to move closer to the vessel wall with each ultrasound cycle. During the third phase of PUT, the shear stress, which is responsible for the bio-effects on the vessel wall, increases much faster than the circumferential stress due to its strong dependence on the distance between the bubble and vessel wall. The greater increase in shear stress will promote the desired bio-effects in the vessel wall, while the smaller increase in circumferential stress will prevent the vessel from rupture. In addition, if similar PUT parameters are used on vessels of different radii but with the same material properties and thickness, the bio-effects may slightly decrease in vessels with smaller radii as opposed to vessels with larger radii due to the decrease in the shear stress values. However, the chances of vessel rupture can be reduced by a greater extent as the circumferential stress, which is more dependent upon the bubble amplitude, will decrease significantly.

This study was an extension of our previous work on PUT.^50^ A 3D FEM-based model was developed in this study to simulate the bubble and blood–vessel interaction during the last phase of PUT, while our earlier study used a 2D axisymmetric FEM model to simulate the bubble and blood–vessel interaction during the first phase of PUT.^50^ PUT is currently in the preclinical stage, and many of the *ex vivo* and *in vivo* studies have demonstrated the bio-effects of PUT on the vessel wall.^47,51,66–68^ The current study on bubble dynamics, bubble–vessel interaction, and induced stresses on vessel wall will be helpful for further improvement in PUT technology. The main challenge of PUT is to choose the proper combination of ultrasound and laser parameters to achieve the desired bio-effect. A higher value of shear stress that may induce stronger bio-effects is desired while the circumferential stress should be below the limit for vessel ruptures at the same time. This study provides further theoretical explanations for cavitation-related shear stress-induced bio-effects observed in our earlier experiments during PUT.^47,51,66–68^ More specifically, we found that the movement of the bubble toward the vessel wall significantly affects the amplitude of the induced stresses. However, the impact on circumferential stress and shear stress is different. The increment in circumferential stress is much less as compared to shear stress when a bubble moves toward the vessel wall. Moreover, if the bubble oscillation amplitude is altered either by changing the confinement or the ultrasound wave parameters, the circumferential stress is more prone to the change than the shear stress.

This study provides insight into the bubble and vessel wall interaction in the last/third phase of PUT, which can be useful for other studies on ultrasound-induced cavitation. However, this study is not without limitations. The first limitation of the study is the use of a smaller vessel radius of only 10 *µ*m and a bubble size of 2 *µ*m for the simulations. In the *in vivo* PUT experiment, the vessel sizes of 100–200 *µ*m are treated and bubble sizes of up to 10–20 *µ*m are present. The results obtained here for the smaller vessel will apply to the larger vessel case also if a similar vessel to bubble size ratio and bubble oscillation amplitude is maintained. However, simulation of a large vessel would have resulted in a better understanding of the interaction between the bubble and vessel wall. The second limitation is assuming the entire process as isothermal with a polytropic index (k) equal to 1. The cavitation process is neither completely adiabatic (k = 1.4) nor completely isothermal (k = 1); the value of k is between 1 and 1.4.^80^ The value of k was assumed as 1 as for other values of k, heat flow module is also required in the model. The third limitation is that the results obtained in this study are not applicable to other studies using contrast agents as cavitation nuclei. The numerical model here does not include the mechanical effects of the contrast agent’s shell.

## V. CONCLUSION

This study was in continuation of an earlier study on PUT, which used the 2D axisymmetric FEM model to simulate the interaction between blood vessels and on-center oscillating bubbles during the beginning phase of PUT.^50^ In this study, a 3D FEM-based model was developed to simulate the interaction between blood vessels and off-center oscillating bubbles during the last phase of PUT. Both the circumferential and shear stresses increased when the bubble was moved toward the vessel wall and decreased when the vessel size was reduced. The shear stress was found to be more dependent on the bubble–vessel wall distance, while the circumferential stress was more dependent on the bubble oscillation amplitude. In addition to the induced stresses, the bubble shape also changed from spherical to ellipsoid during its movement toward the vessel wall, and its oscillation amplitude decreased when it was placed in smaller sized vessels. The finding of a higher increase in shear stress as compared to a less increase in circumferential stress during bubble movement toward the vessel wall explains the enhanced bio-effects observed due to PUT without causing the vessel rupture. The other finding of a less decrease in shear stress than circumferential stress when a bubble was placed in a smaller sized vessel could be helpful for designing future experiments. In addition, the findings regarding the bubble oscillation shape and amplitude help us to better understand the mechanism behind the PUT technology.

## SUPPLEMENTARY MATERIAL

The supplementary material has one figure showing the volume of a 2 *µ*m bubble placed at 0–7 *µ*m away from vessel center inside a 10 *µ*m vessel.

## Data Availability

The data that support the findings of this study are available from the corresponding author upon reasonable request.
